# New Insights into Zika in Infants and Children

**DOI:** 10.3390/tropicalmed7080158

**Published:** 2022-07-30

**Authors:** Sarah B. Mulkey, Roberta L. DeBiasi

**Affiliations:** 1 Prenatal Pediatrics Institute, Children’s National Hospital, Washington, DC 20010, USA; 2 Department of Pediatrics, The George Washington University School of Medicine and Health Sciences, Washington, DC 20052, USA; 3 Department of Neurology, The George Washington University School of Medicine and Health Sciences, Washington, DC 20052, USA; 4 Division of Pediatric Infectious Diseases, Children’s National Hospital, Washington, DC 20010, USA; 5 Department of Microbiology, Immunology and Tropical Medicine, The George Washington University School of Medicine and Health Sciences, Washington, DC 20052, USA

Over the past seven years, the global community of clinicians, researchers, and public health professionals have collaboratively discovered and disseminated knowledge about the congenital Zika virus infection, which was virtually unknown prior to 2015. It is remarkable to reflect upon the progress made in this relatively short time, from the initial emergence of a “new” congenital infection, which was potentially devastating to unborn babies, to our current much deeper understanding of this disease. It is a testament to the many people that have engaged in focused research, clinical care, and advocacy regarding congenital Zika virus infection. This research is being conducted all over the world as we now seek to understand long-term childhood outcomes of Zika virus. From the Zika virus epidemic, many important lessons were learned about fetal infection, the risk to offspring neurodevelopment and importance of health surveillance and global partnerships, which are applicable to many other diseases affecting fetuses and newborns. Due to a heightened awareness of the impact infections could have on the pregnant mother–fetal dyad because of the recent Zika virus epidemic, there was an early recognition of the potential threat to an unborn child of a mother with SARS-CoV-2 infection in pregnancy during the current COVID-19 pandemic. 

During the past seven years, our understanding of the diagnosis and limitations of Zika virus diagnostic testing has improved. We have learned how to evaluate pregnant women, infants, and children for neurologic and other organ system abnormalities associated with Zika virus exposure, and we are now just fully beginning to appreciate the range of long-term neurodevelopmental outcomes associated with prenatal Zika virus infection [1,2,3,4]. In the coming years, we will continue to learn more about how the virus may impact children of school age and beyond. However, despite what we have learned there are still many remaining gaps in our knowledge about Zika virus in infants and children. We need to better understand genetic and environmental risk factors for infection and brain injury severity, find methods to improve neurodevelopment in Zika virus-exposed infants and children who are at risk or have demonstrated developmental delays, we require better laboratory diagnostics to reliably diagnose congenital infections in infants and children, and we ultimately need to prepare for a future epidemic of Zika or other congenital infections that may have serious effects on the developing brain. 

In this Special Issue of *Tropical Medicine and Infectious Disease*, entitled ‘Zika in Infants and Children’, we present an outstanding collection of ten manuscripts that contribute to our enhanced knowledge of Zika virus in infects and children. The manuscripts describe patients from Brazil, Colombia, Honduras, Thailand, the United States (U.S.) Virgin Islands, and from the World Health Organization’s (WHO) Zika Individual Participant Data Meta-Analysis (IPD-MA), which includes data from 28 countries and territories [5]. The themes presented in this Special Issue include adverse pregnancy outcomes, such as birth defects [6], infant and child neurodevelopment [6,7,8], indicators of risk for adverse neurodevelopment [8,9], postnatal Zika virus infection [7], head circumference measurement [10], ocular findings [11], importance of data sharing [5], surveillance systems [5,6,10,12], and a health brigade care model [11,13].

In a cohort of pregnant women with symptomatic Zika virus infection during pregnancy in Colombia, 4.2% of fetuses/infants were found to have Zika-associated brain or eye defects, including microcephaly at birth, which is comparable to other published cohorts [6]. In the study by Mercado-Reyes M. et al., children with Zika-virus-associated birth defects more commonly had adverse neurodevelopmental outcomes in early childhood compared to children without Zika-virus-associated birth defects [6]. In addition to birth defects, early growth parameters seem to be predictive of a developmental delay in children with antenatal exposure to Zika virus and is described in an article by Rose and colleagues [8]. Infant growth parameters of head circumference and length at birth and infant follow-up correspond to a developmental delay and show the importance of accurate head and body measurements of infants at birth and during early childhood [8]. However, it is suggested in the article by Harville and colleagues that head circumference measurement may not be fully accurate and should be interpreted with caution [10]. Despite education on how to accurately measure infant head circumference, the majority of values only included whole-centimeter digits among infants in a microcephaly surveillance study in Honduras [10]. Measurement error may thus impact surveillance data, affecting the ability to detect microcephaly that may be at more mild or borderline values. Postnatal microcephaly can occur in congenital Zika syndrome due to the failure of normal brain growth from injury, so accurate measurements are important as children are evaluated at follow-up visits [14]. Longitudinal measurements of head and somatic growth may be helpful to observe trends over time and correlate these trends with neurodevelopmental outcomes.

Much research has focused on congenital infection for Zika virus infections in infants and children. However, the infant brain experiences important phases of development postnatally and throughout early childhood. In the review article by Drs. Jessica Raper and Ann Chahroudi, emerging clinical evidence supports the hypothesis that Zika virus infection during early infancy can result in negative neurologic consequences [7]. Animal models have helped us to understand the potential risk to young infants with Zika virus infection [7]. Human studies also must include infants and children with early-in-life Zika virus infection, so that we can more accurately understand outcomes in this setting. Large international studies such as the Zika IPD-MA only address prenatal Zika virus infection [15]. Postnatal Zika virus infection studies are more difficult to accomplish, owing to the difficulty in establishing postnatal Zika infection confidently, due to limitations in serologic diagnosis including cross-reactivity of other flaviviruses in endemic areas. Regardless, the evaluation of postnatally Zika virus-infected infants for effects on neurodevelopment should be a future priority, especially if there is a future epidemic.

The effective study of an emerging global infectious disease requires robust international research partnerships. The Zika virus IPD-MA is currently harmonizing data from over 52 cohort studies from 28 countries and territories to determine the risk of adverse pregnancy and infant outcomes, risk on childhood outcomes, and to enable a plan to address a future Zika virus epidemic [5,15]. While this partnership is one on a global level, the Vigilancia de Embarazadas con Zika (VEZ) project in Colombia is at the national level and includes data from three Colombian cities to identify pregnancy, birth, infant, and early childhood neurodevelopmental outcomes for that country [6]. The VEZ project was a collaboration between the Instituto Nacional de Salud of Colombia and the U.S. Centers for Disease Control and Prevention and contributes data to the Zika IPD-MA. Partnerships also help to provide the necessary care for children affected by Zika virus. Recommended evaluations for infants and children with antenatal Zika virus exposure, include evaluations by medical specialists, such as ophthalmology, neurology, infectious disease, audiology, and neurodevelopmental pediatrics, which are often not widely available in all areas where affected children live [1,13]. A health brigade model was developed by a partnership between the U.S. Virgin Islands Department of Health, the U.S. Centers for Disease Control and Prevention, the American Academy of Pediatrics, and the Health Resources and Services Administration (HRSA). This Special Issue contains a report derived from this brigade model, in which 88 children were given comprehensive multi-disciplinary health screenings in 2018 [13]. Among the children evaluated, about one-quarter had visual impairment, confirming the importance of ophthalmological evaluation for all children with antenatal Zika virus exposure [11]. The Zika Health Brigade formed a successful partnership between public health and clinicians to bring care to children in need of evaluations [13]. This successful brigade model has been used to provide care to other Zika-affected populations and may be effective for providing necessary multi-specialty evaluations to other areas where women and children are affected by other environmental or infectious threats.

Some areas with consistent knowledge gaps are related to an understanding of differences in pregnancy and infant outcomes. Prenatal viral infection is complex, with many factors likely impacting outcomes, including viral virulence, the timing of infection, as well as host genetic responses and environmental factors. In a review article, Drs. Kousa and Hossain describe how prenatal viral infection can lead to a spectrum of adverse neurodevelopmental conditions or fetal demise and how phenotypic variability between cases may arise following prenatal viral infections [9]. Their manuscript lays the groundwork for applications of this knowledge to other infectious agents that may pose a threat to the developing brain. These issues are applicable in a presented case report of discordant clinical outcomes in a monozygotic dichorionic–diamniotic twin pregnancy, with one twin having severe Zika-associated birth defects and the other twin having none [12]. It is likely that genetic and intrauterine environmental factors contribute to the differing phenotypes described in this case report [9].

While the epidemic circulation of Zika virus has ended for now, we must continue to expand our knowledge about the women whose pregnancies were impacted by Zika virus infection and their infants and children who are now growing up, so that we can best support their current and future needs. We must also remain vigilant and maintain our preparedness to care for individuals impacted by future epidemics. Travelers to geographic locales with Zika virus transmission, such as Thailand, should remain aware of the potential for Zika virus infection and use precautions to avoid mosquito bites [16]. Zika virus is still circulating, but at much lower levels, and with concomitantly lower levels of risk of transmission. When evaluating a fetus, infant, or child with congenital defects or developmental delays consistent with Zika virus infection, testing for this virus should be considered based on an epidemiological link to a place of potential exposure. Finally, the global community needs to continue to work together to support surveillance efforts to detect any signal of increased Zika virus infections and/or other novel infectious threats to the mother, fetus, infants, or to children, that may pose a potential negative impact on child development. We thank the authors who contributed to this Special Issue for enhancing our knowledge on Zika virus in infants and children.
